# Untangling the effects of within-host and between-host natural selection in viruses with applications to evolution of HIV-1 co-receptor usage

**DOI:** 10.1098/rsif.2025.1012

**Published:** 2026-09-09

**Authors:** Kieran O. Drake, Vinicius Bonetti Franceschi, Fabrícia F. Nascimento, Erik Volz, on behalf of the PANGEA Consortium

**Affiliations:** MRC Centre for Global Infectious Disease Analysis, School of Public Health, Imperial College London, London, UK

**Keywords:** evolution, phylodynamics, coalescent, pathogen fitness, within-host, between-host, HIV, co-receptor switching, BiSSE

## Abstract

The fitness of a pathogen can be characterized at multiple levels corresponding to different life cycle stages. Typically, epidemiological studies for measuring pathogen fitness compute a single selection coefficient for pathogen variants that encompasses the entire lifecycle. However, evolution towards greater fitness within hosts can potentially compromise transmissibility. To address this, we develop a population-genetic model and software for the inference of multi-level selection coefficients. Our approach builds on population-genetic models previously applied to macro-evolution, whereby speciation and extinction rates are state-dependent. We relate this state dependence to within- and between-host selection. Evolution of beneficial phenotypes within hosts may occur rapidly (higher ‘speciation’), but reduce rates of dispersal between hosts (higher ‘extinction’). This new coalescent-based method improves on existing multi-state speciation and extinction models by accounting for highly nonlinear population dynamics and variable sampling through time, making it suitable for epidemiology. We characterize the accuracy of this method using birth–death and coalescent simulations before analysing a real dataset of HIV-1 genetic sequences. We quantified the multi-level relative fitness underlying HIV-1 ‘co-receptor switching’. In accordance with previous observations, we estimate that the relative transmissibility of X4-tropic virus is 9%, while within-host switching to X4-tropism is approximately 7-fold higher than switching to R5-tropism, indicating a strong within-host and between-host trade-off of co-receptor usage.

## 1. Introduction

Microbial evolution often features episodic natural selection that stems from competing selective pressures in fluctuating environments. The life cycle of a pathogen exhibits such a fluctuating environment, and requires the successful completion of multiple steps: crossing tissue barriers, evading innate immunity, binding to a target cell, replicating within host cells, evading adaptive immunity, and shedding infectious particles such that the cycle may repeat. The selective pressures at each stage can conflict. For example, pathogen phenotypes optimized for one step (such as immune evasion) may perform poorly at others (like cell binding). Additionally, the successful spread of viruses such as HIV-1 depends on a transmission–virulence fitness trade-off: the pathogen must replicate sufficiently within the host to be transmitted, yet not causing rapid progression to severe illness or death that would limit opportunities for onwards transmission [1–4]. Therefore, natural selection within hosts necessarily involves compromising some steps of the infection cycle to advance others. In order to quantify such trade-offs, we have developed a new model that enhances existing methods used in ecological studies [5] to make them suitable for epidemiology by accounting for highly nonlinear population dynamics and variable sampling through time.

Within-host and between-host evolutionary compromises are well documented across diverse pathogens. The evolution of antimicrobial resistance often comes at the cost of reduced replicative fitness within host cells. Similarly, mutations enabling escape from adaptive immunity can impair receptor binding capacity [6]. A particularly illustrative example, which we examine in detail below, concerns HIV-1 co-receptor switching [7–9], such that different viral phenotypes experience varying selective advantages during the course of a single host infection. The use of the R5 co-receptor by viral variants to enter host cells is predominant at transmission and during all stages of infection. However, HIV-1 evolves during the course of infection to switch to the X4 co-receptor (or both co-receptors, i.e. dual tropic/R5X4) in up to 50% of individuals [7,9], which is associated with a poorer baseline clinical status [10] and faster progression to AIDS [11,12]. Although early studies suggested that R5 viruses were preferentially selected at transmission, as a result of a single or multiple leaky ‘gatekeepers’ [13,14] (i.e. *in vivo* biological mechanisms that restrict the transmission of X4 viruses more efficiently than R5 viruses), later transmission cluster analysis demonstrated that individuals infected by X4 viruses regularly transmit X4/R5X4, suggesting a stochastic process (random transmission hypothesis) [15,16]. We address this question formally, and our findings support the existence of reduced transmissibility of X4 virus while indicating that limited transmission of X4 virus can still occur owing to stochastic effects.

In quantitative terms, it is impossible to describe the fitness of a given phenotype using a single selection coefficient in the presence of multi-level selection. While multiple selection coefficients are needed to fully describe fitness variation across different environments, epidemiological studies have typically collapsed these into single estimates of variant fitness [17,18]. Despite the ubiquity of episodic selection in pathogens, there has been relatively little progress on methods to estimate multi-level selection coefficients of genomic variants. This is a challenging inference because of the large difference in timescales governing within-host pathogen replication and between-host transmission. Evolutionary rates within hosts are typically much higher than those measured between hosts, and the replication cycle of a pathogen between cells can be many orders of magnitude faster than the infection cycle between hosts. Another difficulty concerns the type of data available. Epidemiological surveillance data typically provide only one or a handful of sequences per host, but assessing the within-host fitness and transmissibility of particular variants would ideally make use of many sequences from hosts taken over the course of their infection which would itself be drawn from a population-based sample of cases. While deep sequencing data are enabling investigations into within-host microbial evolution [19,20], such data are typically only collected at a single time point for each host.

In this paper, we present a general-purpose method for inferring multi-level selection coefficients for epidemiological sequence data. Our approach is similar to population-genetic models previously applied in the macro-evolution setting, where multi-level pathogen selection parallels state-dependent speciation and extinction over evolutionary timescales [5]. In phylodynamic terms, competing within-host and between-host selection creates analogous state dependence in speciation and extinction rates, but compressed into epidemiological timescales. Evolution of beneficial phenotypes within hosts may occur rapidly (higher ‘speciation’), but face reduced rates of dispersal between hosts (higher ‘extinction’). The key insight that enables inference of multi-level selection coefficients is that fitness depends on phenotypic state, which changes over the phylogeny of the pathogen. Estimation of selection coefficients is thus coupled to reconstruction of ancestral phenotypes given the observed traits in sampled lineages.

Some limitations of previously developed state-dependent selection models preclude their use for analysis of infectious diseases. On the short timescales of pathogen evolution, epidemiological dynamics need to be taken into account. Epidemic dynamics often lead to highly nonlinear changes in effective population size that cover many orders of magnitude. Furthermore, in contrast to studies of macro-evolutionary processes, sampling of microbes is highly distributed through time, and pathogen phylogenies often consist of a mixture of sampled lineages near the present and close to the most recent common ancestor. Sampling through time is often non-random, and the rate of sampling can vary drastically and unpredictably over time, since sampling is often non-systematic and responsive to emerging public health incidents.

In this study, we develop a coalescent-based method for inferring multi-level selection that accounts for highly nonlinear population dynamics and variable sampling through time. Our approach provides a general-purpose tool for inference of multi-level selection coefficients. It can be applied to any dataset where a time-scaled phylogeny can be estimated and where the phenotype (e.g. drug resistance, immune escape or co-receptor usage) is known for each sample. This method is most appropriate for situations where one pathogen genome is sampled per host. It is optimized for analyses of pathogen genomes by automatically estimating effective population size over time ($N_{e}(t)$), adjusting for nonlinear-population dynamics, allowing for heterochronous sampling and conditioning on known sample dates.

## 2. Methods

### 2.1. Model overview

We have developed a model to estimate values for the ratios of the between-host transmission rates for two variants and within-host mutation rates between those variants. We define the former ratio as ω and the latter as α, as described in §2.2. These values are estimated using a structured coalescent maximum likelihood (ML) method. The model requires several key assumptions: the generation time is constant between variants; there is negligible within-host genetic diversity; input phylogenies are constructed from population-based random samples and at most one sequence per host. Two options are available for computing the estimates of α and ω: a coalescent likelihood and an augmented-coalescent likelihood [21]. The latter combines the former with a likelihood of sampling each variant, but this relies on the assumptions of random sampling and mutation–selection balance, as described in §2.2.1. We assess the relative performance of these two methods using simulated data (§§2.4 and 3.1). The model is available as an R package, *musseco* [22]. Figure S1 in the supplementary material shows an overview of the workflow of *musseco*. Further details of the model implementation and required inputs are in §2.6.

### 2.2. The binary-state mutation and transmissibility model

In analogous fashion to the binary-state speciation and extinction (BiSSE) model [5], we specify the dynamics of two variants with different fitness at separate timescales. Our model is premised on the coevolution of two variants A and V, an ancestral type and variant respectively, such that within hosts, V has a selective advantage, while also having lower transmissibility between hosts.

The variants have different rates of transmission between hosts, with time-dependent *per capita* rates $\beta_{a}(t)$ and $\beta_{v}(t)$. We parametrize the relative fitness of variant V using

(2.1) $$\begin{array}{rl} \beta_{v}(t) & =\omega \beta_{a}(t), \end{array}$$

and assuming a fitness penalty for V such that ω<1. We sometimes also refer to the *selection coefficient* for V defined as s=ω−1 and require that −1<s<0. We also assume that the generation time (mean time between successive infections) is the same between variants.

This model will require that each infected host at each time point is colonized by either A or V and that when de novo mutation of V occurs, there is no interval of coinfection with both variants. This model is therefore premised on within-host genetic diversity being negligible. We parametrized the increased within-host fitness of V in terms of an imbalance between the rate of mutation from V to A and vice versa. The mutation rates in a single host from A to V and from V to A are denoted $\mu_{av}$ and μ, respectively. Our model is premised on increased mutation from A to V by a factor α:

(2.2) $$\begin{array}{r} \mu_{av}=\alpha \mu , \end{array}$$

such that α>1. Describing multi-level selection therefore requires estimation of two parameters, ω and α.

The number of infected hosts with each variant over time is $Y_{a}(t)$ and $Y_{v}(t)$, and the total size is $Y(t)=Y_{a}(t)+Y_{v}(t)$. Unless otherwise stated, we will assume that the variants are in a mutation–selection balance (cf. §2.2.1), such that the proportion of the ancestral type $p_{a}=Y_{a}(t)/Y(t)$ is constant over time.

**Figure 1.**
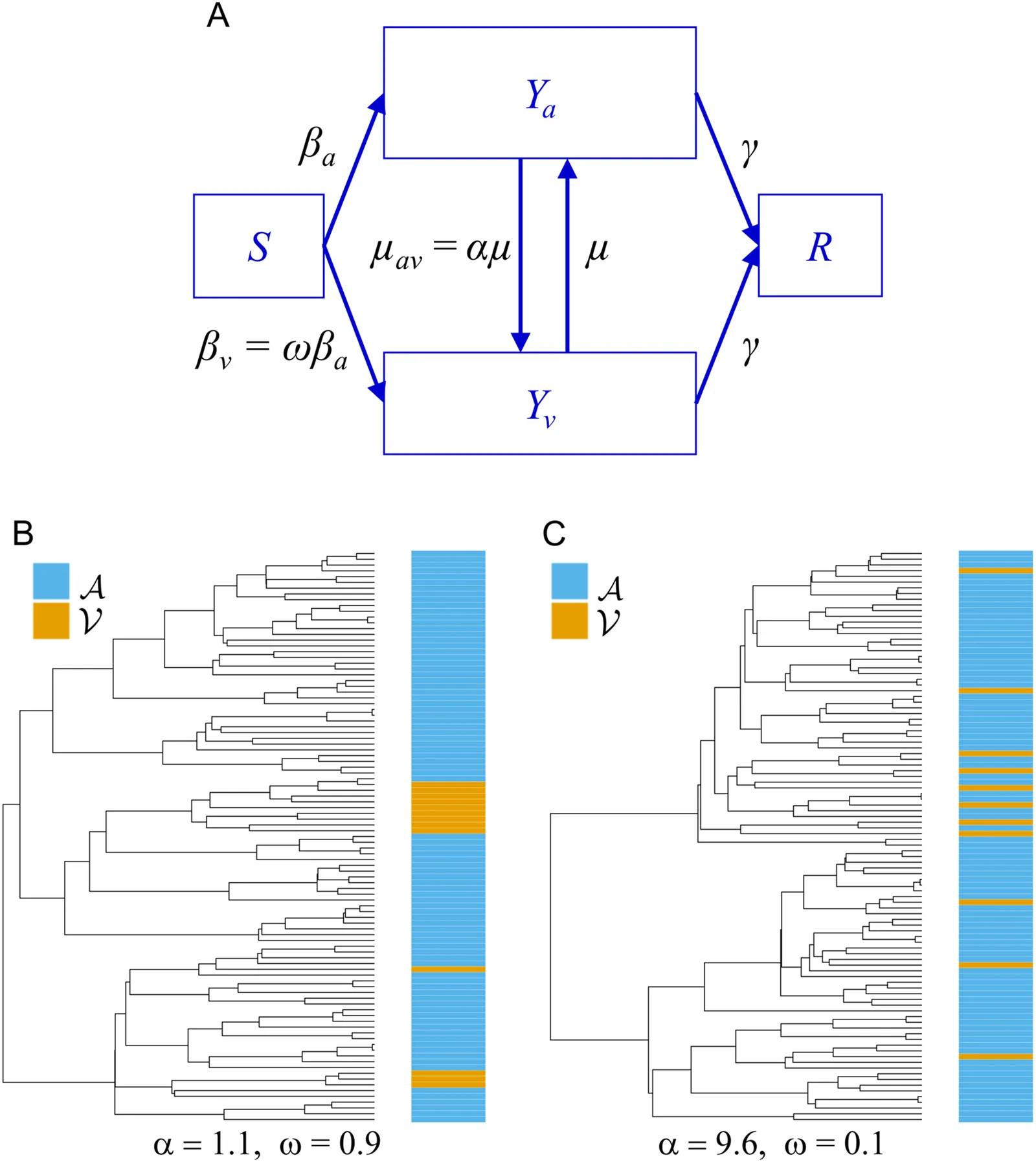
Binary-state mutation and transmissibility model. (A) Illustrative epidemiological compartmental model considering multi-level fitness. The model includes two variants, A and V, which have different between-host transmission rates, with ratio ω, and asymmetric within-host mutation rates with ratio α. (B,C) Genealogies with identical mutation–selection balance of V (≈15%), yet with different cladistic patterns stemming from different within- and between-host selection. Phylogenies with 100 tips were simulated with *diversitree* [23] and plotted using *ggtree* R package [24]. (B) Weak within- and between-host selection. The relative transmissibility of V is 90% (ω=0.9) and α=1.1. (C) Strong within- and between-host selection. The relative transmissibility of V is 10% (ω=0.1) and α=9.6.

A compartmental model diagram summarizing the coevolution of A and V is shown in figure 1. In §§2.2.1 and 2.3.1, we show how these parameters determined a mutation–selection balance between variants and how to account for epidemiological dynamics by appropriate choice of functional forms for Y(t) and $\beta_{x}(t)$.

Figure 1 also illustrates genealogies simulated under different scenarios of strong and weak within-host and between-host selection. In the weak-selection scenario, clades of V have longer persistence times and larger size, but under the constraint of a given mutation–selection balance, such a variant has correspondingly weak within-host selection leading to infrequent de novo occurrences within hosts. By contrast, under strong within- and between-host selection, V occurs frequently throughout a genealogy, but clades of V rapidly go extinct.

#### 2.2.1. Mutation–selection balance

If the variant and ancestral types are in equilibrium (mutation–selection balance), it is possible to construct a likelihood of sampling proportions that are of the ancestral type ($p_{a}$) and variant type ($p_{v}=1-p_{a}$) assuming random sampling. This likelihood can be used in combination with the structured coalescent likelihood (cf. §2.3) to gain additional precision when estimating selection coefficients, although the conditions of random sampling and mutation–selection balance should be evaluated first. We define this combined likelihood as the ‘augmented-coalescent’ likelihood. Further details can be found in §S1 in the supplementary material.

Here, we solve for $p_{a}$, which is a function of α, ω and time, by solving a set of detailed balance equations involving transmission, death (or recovery) and mutation. This model is premised on the death (or recovery) rate having a constant *per capita* rate γ. The corresponding rates are:

**Table:** 

	ancestral	variant
transmissions:	$+\beta_{a}(t)Y_{a}(t)$	$+\beta_{v}(t)Y_{v}(t)$
		$=+(1+s)\beta_{a}(t)Y_{v}(t)$
		$=+\omega \beta_{a}(t)Y_{v}(t)$
deaths:	$-\gamma Y_{a}(t)$	$-\gamma Y_{v}(t)$
mutations:	$-\mu_{av}Y_{a}(t)+\mu Y_{v}(t)$	$-\mu Y_{v}(t)+\mu_{av}Y_{a}(t)$
	$=-\alpha \mu Y_{a}(t)+\mu Y_{v}(t)$	$=-\mu Y_{v}(t)+\alpha \mu Y_{a}(t)$

The detailed balance condition in terms of $p_{a}$ can be expressed as

(2.3)paβa(t)=γpa+μαpa−μ(1−pa)(2.4)and(1−pa)ωβa(t)=γ(1−pa)+μ(1−pa)−μαpa.

Combining these equations and solving for $p_{a}$ yields

(2.5) $$\begin{array}{rl} 0 & =p_{a}(\gamma (1-p_{a})+\mu (1-p_{a})-\mu \alpha p_{a})-(\omega (1-p_{a}))(\gamma p_{a}+\mu \alpha p_{a}-\mu (1-p_{a})). \end{array}$$

This quadratic equation in $p_{a}$ has at most one root in the unit interval given the positive domain of all other parameters.

### 2.3. The structured coalescent and modelling variant coevolution

To model gene genealogies generated by the binary-state coevolution model, we use the structured coalescent framework previously developed [25,26]. This coalescent model specifies the rates of lineage migration and coalescence in terms of the corresponding population dynamics comprising three deterministic matrix-valued functions of time and the state of the system with m demes:

— Births: $F_{1:m,1:m}(t,Y)$. In this study, this represents between-host transmission rates for different pathogen variants.
— Mutation: $G_{1:m,1:m}(t,Y)$. In this study, this represents within-host mutation rates.
— Deaths: $\Gamma_{1:m}(t,Y)$. These terms may also correspond to recovery or removal from the infectious population in epidemiological models.

We order m=2 demes corresponding to the coevolving variants: D=(A,V). Then, the *birth* matrix represents dispersal rates to new hosts and depends on the relative transmissibility parameter ω:

(2.6) $$F(t)=\left[\begin{array}{cc} \beta (t)p_{a}Y(t) & 0\\ 0 & \omega \beta (t)(1-p_{a})Y(t) \end{array}\right],$$

where β(t) is the time-varying *per capita* transmission rate and Y(t) is the total number of infected hosts. We show below how β(t) and Y(t) are derived in terms of the effective population size over time. These birth rates appear on the diagonal since a host colonized with a particular variant may only generate new infections of the same type. By contrast, mutation terms appear on the off-diagonal and generate the simultaneous loss of a lineage of one type (A or V) and the gain of the other type:

(2.7) $$G(t)=\left[\begin{array}{cc} 0 & \alpha \mu p_{a}Y(t)\\ \mu (1-p_{a})Y(t) & 0 \end{array}\right].$$

Finally, death rates determine the generation time $T_{g}=1/\gamma$ of the process and allow translation of genealogical time to calendar time:

(2.8) $$\Gamma (t)=\left[\begin{array}{c} \gamma Y_{a}(t)\\ \gamma Y_{v}(t) \end{array}\right]=\left[\begin{array}{c} \gamma p_{a}Y(t)\\ \gamma (1-p_{a})Y(t) \end{array}\right],$$

where $T_{g}$ is the assumed constant between variants.

In the supplementary material, we review how the likelihood of a tip-labelled genealogy is computed conditional on the epidemiological history specified by M=(F(t,Y),G(t,Y),Γ(t,Y)). With M defined, the population size of each deme k can be computed by solving the system of ordinary differential equations:

(2.9) $${\dot{Y}}_{k}=-\Gamma_{k}(t)+\sum_{l=1}^{m}(F_{lk}(t)+G_{lk}(t)-G_{kl}(t)).$$

#### 2.3.1. Effective population size

To adjust for variation in the coalescent rate that is unrelated to the coevolution of A and V (e.g. owing to epidemiological dynamics), we calibrate β(t) and Y(t) so that the total coalescent rate (averaged over all demes) matches an empirical estimate that is obtained by an independent phylodynamic analysis. Specifically, suppose that we are provided with an estimate of $N_{e}(t)$ for the entire gene genealogy that neglects population structure in the form of the co-circulating variants. Our software implementation will estimate $N_{e}(t)$ using a non-parametric ML method by default [27] (cf. §2.6), but in principle, any method can be used and provided as an input to our algorithm. Note that the scale of Y(t) is arbitrary, because the structured coalescent is overdetermined, and many different forms of β(t) and Y(t) would yield the same $N_{e}(t)$. We need only to derive a definition such that changes in Y(t) yield identical changes in the pre-estimated $N_{e}(t)$. Our approximation to Y(t) is premised on the following:

— Without loss of generality, $\beta_{a}(t)=1$, and transmissions $\phi_{a}(t)$ per unit time from A will be equal to population size $Y_{a}(t)$. For V, we will use $\beta_{v}(t)=\omega$, and transmissions $\phi_{v}(t)$ will be: $\phi_{v}(t)=\omega Y_{v}(t)$.

— The coalescent rate for a pair of lineages in a deme x can be derived in various ways. Here, we use the coalescent method derived by Volz [25], which uses the variance of the offspring distribution as a function of the time-dependent birth rate to define a cumulative hazard of coalescence as $\Lambda_{2}(t)=\sum_{j=1}^{\left\lfloor F(t)\right\rfloor }\sigma_{M}^{2}(j)/\overset{―}{Y}(j)$, where $\sigma_{M}^{2}(j)$ is the variance of offspring, $\overset{―}{Y}(j)$ is the population size, both at the *j*th birth event, and F(t) is the cumulative number of births prior to time t. This is similar to Cannings-type models, but sums over individual birth events. After accounting for non-constant birth rates, the coalescent rate is shown to be: $\lambda_{x}(t)=2\phi_{x}(t)/Y_{x}(t{)}^{2}$. This coalescent model enables us to define coalescant rates for demes A and V:

λa(t)=2ϕa(t)/Ya(t)2(2.10)=2paY(t)andλv(t)=2ϕv(t)/Yv(t)2(2.11)=2ω(1−pa)Y(t).

— Given the binomial probabilities $p_{x}^{2}$ of selecting two lineages from the same deme x, the approximate pairwise coalescent rate for the population as a whole will be

(2.12)λ(t)=pa2λa(t)+(1−pa)2λv(t)(2.13)=2paY(t)+(1−pa)2ωY(t),

where the last line is obtained by substituting expressions from equations (2.10) and (2.11).

To solve for Y(t), we equate λ(t) (equation (2.13)) to $\iota /N_{e}(t)$, where ι is an additional scaling parameter, which is estimated to improve the overall estimate of the coalescent rate. This yields

(2.14) $$\begin{array}{rl} Y(t) & =\frac{2N_{e}(t)}{\iota }\left(p_{a}+(1-p_{a})\omega \right). \end{array}$$

In practice, we observe that estimates of ι are very close to one (cf. §3.1), indicating that equation (2.14) is a good approximation in many situations.

### 2.4. Simulations

We used three methods, which support genealogical simulations in structured populations, *Coalescent.jl* [28], *TiPS* [29] and *diversitree* [23], to simulate trees in which we know the true values of the relative between-host fitness (ω) and relative within-host fitness (α) to test the new coalescent model. Two of these simulators, *Coalescent.jl* [28] and *TiPS* [29], are based on the structured coalescent and require an epidemiological model describing the dynamics of infectious individuals. The *diversitree* method is based on a birth–death model and has previously been used to study the multi-state speciation and extinction process.

We simulated a broad range of values for α and ω (supplementary material, tables S1– S3), covering a diverse range of biological systems, and encompassing the values that we estimate for the HIV-1 co-receptor switching (§3.3).

#### 2.4.1. Epidemiological model

To carry out simulations with *Coalescent.jl* and *TiPS*, we developed a mathematical epidemiological model to describe the dynamics of ancestral and variant states. This model was defined as two ordinary differential equations describing the number of infectious individuals carrying sequences with the ancestral state $Y_{a}(t)$ and the number of infectious individuals carrying sequences with the variant state $Y_{v}(t)$:

(2.15) $$\begin{array}{rl} {\dot{Y}}_{a}(t) & =\beta Y_{a}\left(1-\frac{Y}{K}\right)+\mu Y_{v}-\mu_{av}Y_{a}-\gamma Y_{a}\\ \text{and}\;{\dot{Y}}_{v}(t) & =\beta (1+s)Y_{v}\left(1-\frac{Y}{K}\right)-\mu Y_{v}+\mu_{av}Y_{a}-\gamma Y_{v}. \end{array}$$

The rate of new infections was defined as logistic growth controlled by the parameter β for $Y_{a}(t)$ and β(1+s), where s is the selection coefficient, for $Y_{v}(t)$. The carrying capacity was defined by the parameter K and was fixed at 10 000 individuals. Infected individuals recover at the rate γ.

Individuals containing the ancestral state, $Y_{a}(t)$, mutate to the variant state, $Y_{v}(t)$, at a rate $\mu_{av}$, and individuals carrying the variant state could revert to the ancestral state at a rate μ.

This is a forward simulation model used for validation of our new method.

#### 2.4.2. Genealogical simulations

The parameter values used to simulate genealogies with *Coalescent.jl* (v0.1.4) and *TiPS* (v1.2.3) are described in table S1 in the supplementary material. Both methods employ Gillespie's direct algorithm with time-varying coalescent and migration rates. We obtained the value of the transition rate from $Y_{a}$ to $Y_{v}$ ($\mu_{av}$) using the theoretical equation for the mutation–selection balance using fixed values for the proportion of individuals in the ancestral state, the molecular clock rate (here, also defined as the transition rate from $Y_{v}$ to $Y_{a}$) and the relative transmission fitness (ω=1+s).

To simulate trees with *Coalescent.jl* and *TiPS*, we also provided sampling dates randomly sampled in the past 5 years using the Julia programming language. We then used the same sampling dates from *Coalescent.jl* trees to simulate trees using the *TiPS* R package.

For *diversitree* (v0.10.1) simulations, we analysed different scenarios of population dynamics with a high and low $R_{0}$ (basic reproduction number). Note that $R_{0}$ depends on the proportion of ancestral state and the birth rates. Each combination of parameter values analysed with *diversitree* had a different birth rate, but we grouped the results into two different values of $R_{0}$: a high value of approximately 1.85 and a low value of approximately 1.22 (see supplementary material, table S2, for a complete list of parameter values and the $R_{0}$ for each combination of parameter values used with *diversitree*).

For all combinations of parameter values tested, we also sampled a total of 500 or 1000 individuals to understand how the sample size would influence the estimate of parameters.

#### 2.4.3. Simulation and analysis of genetic sequences

We carried out an additional set of simulations to mimic the observed HIV data described in §2.5. We used *Coalescent.jl* with the same epidemiological model as described in §2.4.1 to simulate genealogies in which trajectories were simulated for 29 or 45 years. For a list of the parameter values used, see table S3 in the supplementary material. We simulated genealogies with 4056 tips with sampling time from the past 4 years (which was observed for the Phylogenetics and Networks for Generalised Epidemics in Africa (PANGEA) dataset, see §2.5). These simulated genealogies will be referred to as ‘true trees’.

We simulated sequence alignments of 105 bp and 2371 bp, based on the observed alignment length for the empirical dataset, using the simulated true trees and the program *AliSim* [30] to understand the impact of sequence alignment length on the estimation of relative fitness α and ω. We simulated sequence alignments without gaps or recombination that started from random starting sequences. We then estimated ML trees using the simulated sequence alignments and the program *IQ-TREE* (v2.4.0) [31], followed by the estimation of time-scaled trees using the strict clock implemented in *treedater* (v.0.5.3) R package. The substitution model and parameters used to simulate the sequence alignments are given in §S1.4 in the supplementary material.

#### 2.4.4. Statistical inference

Fitness parameters ω and α were estimated by ML using prior knowledge of the generation time (equivalent to 1/γ) and the molecular clock rate of evolution, which we assume is equivalent to the reversion rate μ. Inference was carried out using both the coalescent and the augmented-coalescent likelihoods as described in §2.2.1. Approximate confidence intervals were computed assuming asymptotic normality of the maximum likelihood estimate (MLE) and computing standard errors from the observed Fisher information (negative Hessian of the log likelihood).

Performance of the estimator is summarized by relative error ($\left\|(\hat{X}-X)/X\right\|$) and the coverage probability of the 95% confidence interval.

### 2.5. Multi-level evolution of HIV-1 co-receptor tropism

In order to disentangle the effects of within- and between-host selection related to HIV-1 co-receptor usage, we used data from a high-prevalence setting in sub-Saharan Africa. The HPTN 071 study, commonly referred to as Population Effects of Antiretroviral Therapy to Reduce HIV Transmission (PopART), was a large-scale community-based trial conducted in South Africa and Zambia between 2013 and 2018. Its primary aim was to assess the effect of a universal test-and-treat strategy on population-level HIV incidence. During the study period, ≈5% (n>48000) of the total population of >1 million people were surveyed, including blood samples, annually for 3 years [32]. The HPTN 071-2 PopART Phylogenetics Ancillary study used samples from HIV seroconverters within the PopART cohort from Zambia only and HIV-positive samples collected from health clinics across 9 of the 12 Zambian PopART communities, which were then subjected to sequencing and genome assembly processing as previously described [33]. Access to HIV-1 genomes and appropriate metadata was granted by the PANGEA-HIV 2 Steering Committee after the acceptance of a concept sheet proposal.

#### 2.5.1. Data curation and phylogenetic tree reconstruction

HIV-1 consensus sequences were subtyped using *COMET* v2.4 [34], retaining the first sequence per individual and focusing on subtype C (n=5460). After retaining only ART-naive individuals, whole genome sequences were aligned to the HXB2 reference using *MAFFT* v7.505 [35] and subjected to quality control to remove recombinants, hypermutated sequences and short alignments, yielding 4249 sequences (see additional curation details in supplementary material, §S1.5.1).

The *env* gene and V3 loop regions were extracted, with alignment sizes of 2371 and 126 base pairs, respectively. The analyses focused on *env* (with variable loops masked following recommendations from Ratmann *et al.* [36]), as simulations indicated insufficient information in short V3 alignments for reliable inference of the α and ω parameters. A ML phylogeny was inferred using *IQ-TREE* v2.2.2.6 [31] under a general time-reversible model [37] with a free-rate model for rate heterogeneity across four categories (R4) selected by *ModelFinder* v2.2.2.6 [38], followed by additional curation and re-estimation with ultrafast bootstrap support (see details in supplementary material, §S1.5.1). Time-scaled trees were reconstructed using an additive uncorrelated clock model [39] implemented in *treedater* v0.5.3 [40] with 10 maximum iterations. A sub-sampled version of the time-scaled phylogenetic tree is shown in figure S14 in the supplementary material as an example.

#### 2.5.2. Co-receptor tropism and coalescent analyses

V3 loop nucleotide sequences were used to infer co-receptor usage via *WebPSSM* server and a position- and subtype-C-specific SINSI scoring matrix [41,42]. The distribution of *WebPSSM* scores, trends of co-receptor usage over time, distribution of CD4 counts and ranges for each predicted co-receptor and the ratio of predicted R5 versus X4/R5X4 were summarized. For the BiSSECo analyses, we used the time-scaled *env* phylogeny (n=4056 tips from February 2014 to June 2018), treating the X4/R5X4 trait as variant and R5 as ancestral. Model parameters were estimated under both an augmented-coalescent likelihood and a purely coalescent likelihood. The generation time ($T_{g}$) was defined as 1/γ, using the same γ value as in the simulations (supplementary material, table S1). The molecular clock rate was fixed to the *treedater* estimate ($3.2\times 10^{-3}$ nucleotide substitutions per site per year). Effective population size trajectories were inferred using *mlesky* v0.1.7 [27], with smoothing optimized by cross-validation and uncertainty assessed via parametric bootstrap (see details in supplementary material, §S1.5.2).

### 2.6. Implementation

We have implemented the binary-state coalescent model as an open source R package *musseco* [22,43] available at http://emvolz-phylodynamics.github.io/musseco along with a tutorial. The *musseco* package includes a function, fitbisseco, that estimates multi-level selection coefficients by ML and will estimate effective population size automatically if an estimate is not provided. The fitbisseco function requires a number of inputs in order to estimate the fitness parameters. A time-scaled phylogeny is required and can be estimated, for example, using the *treedater* R package [40]. This will also estimate the molecular clock rate of evolution, μ, which is another required input. Pathogen phylogenies are assumed to be reconstructed from population-based random samples of pathogen genomes and at most one sequence per host. The effective population size over time, $N_{e}(t)$, can also be input, although if it is not provided then it will be estimated with the *mlesky* R package [27] using the *skygrid* model [44] by default. This estimate of $N_{e}(t)$ is also provided in the returned BiSSECo fit using the fitbisseco function in the *musseco* R package [22]. Sample states, i.e. ancestral or variant, are also required to be defined for tips in the time-scaled phylogeny and included as an input to the fitbisseco function. An estimate of the generation time, $T_{g}$, is also required. Initial estimates of α, ω and Y(t) scaling parameter are also required as starting points for the estimation process, which is described in the supplementary material.

## 3. Results

### 3.1. Simulations

**Figure 2.**
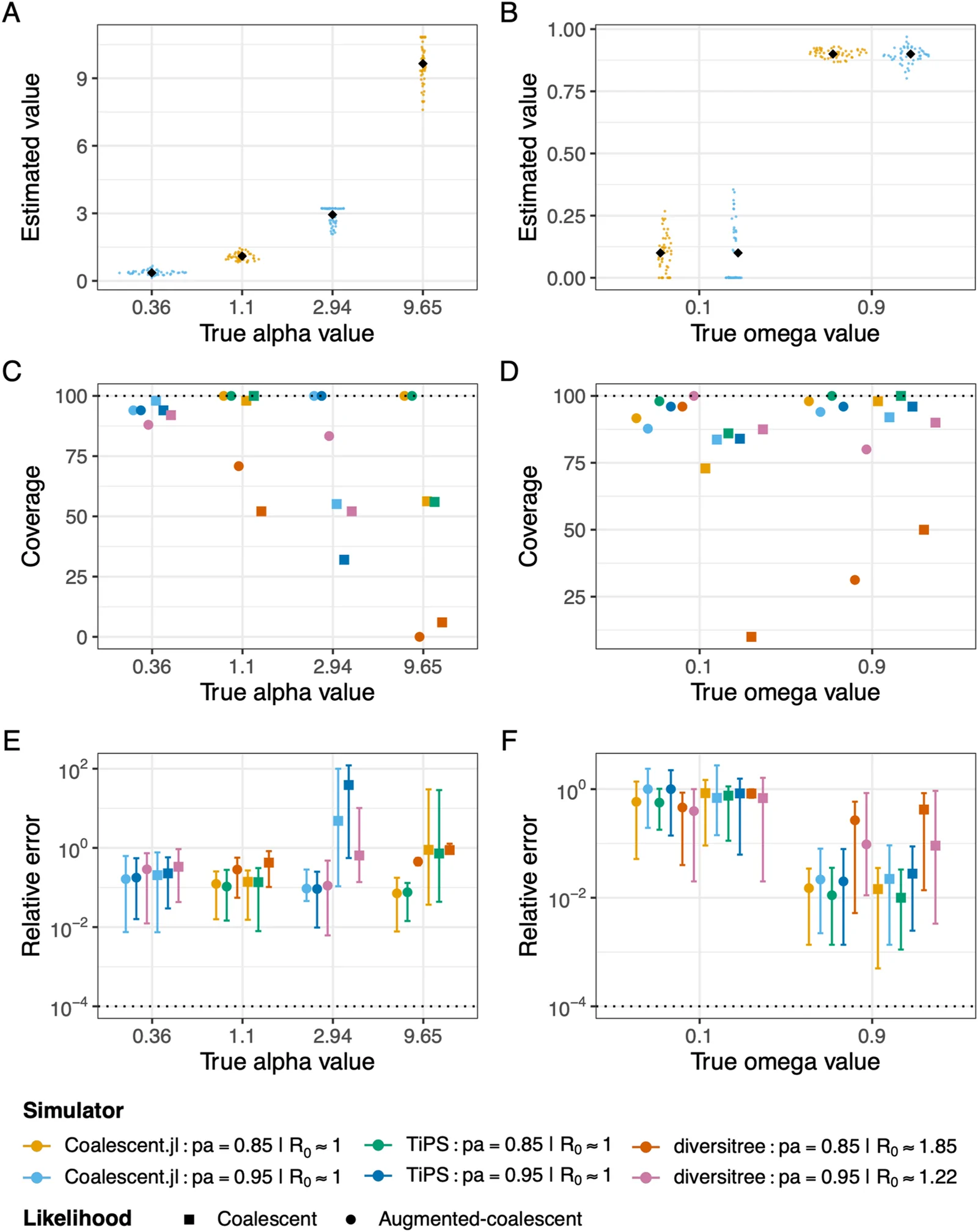
Simulation results for sample size of 1000 tips. Sina plots showing the true and estimated values for (A) α and (B) ω for simulations carried out with *Coalescent.jl* and using the augmented-coalescent likelihood only. Black diamond shapes in the plot are the true values. Coverage defined as the percentage of replicates that contained the true (C) α or (D) ω values within the 95% credible interval for trees simulated with *Coalescent.jl*, *TiPS* and *diversitree*. Relative error for (E) α and (F) ω estimates. The relative error (y-axis) represents the computed median and the 0.025 and 0.975 quantiles for all replicates. Depending on the combination of parameter values, the proportion of individuals carrying the ancestral state ($p_{a}$) could be 0.85 or 0.95. For the *diversitree* analyses, we grouped the values of $R_{0}$ into two groups by averaging the observed $R_{0}$ to 1.22 and 1.85 (see supplementary material). For the *TiPS*/*Coalescent.jl* analyses, we sampled at equilibrium and $R_{0}$ values are expected to be close to 1.0. Fifty simulation replicates were generated for each combination of parameter values.

Across most simulation studies, the augmented-coalescent outperformed the coalescent model that did not include a sampling likelihood based on mutation–selection balance. While the augmented-coalescent MLE generally had good precision (figure 2A), the approximate confidence intervals did not always cover the true value, especially for birth–death simulations with high $R_{0}$.

#### 3.1.1. Coverage

When estimating α, coverage was generally close to or equal to 100% for analyses carried out with *Coalescent.jl* or *TiPS*. However, when the true α value was 2.94 or 9.65, the augmented-coalescent likelihood performed better than the coalescent likelihood (figure 2; supplementary material, figure S5). For trees simulated with *diversitree*, it was clear that coverage was higher when $R_{0}$ was low, and the true value of α was 0.36 or 2.94. When $R_{0}$ was high, coverage was close to zero when the true value of α was 9.65. In general, the augmented-coalescent likelihood also performed better than the coalescent likelihood (figure 2; supplementary material, figure S5).

When estimating ω, coverage was generally close to or equal to 100% when the true ω value was 0.9 for trees simulated using *Coalescent.jl* or *TiPS*. In most cases, the augmented-coalescent likelihood performed better than the coalescent likelihood (figure 2; supplementary material, figure S5). For analyses carried out with trees simulated with *diversitree*, we observed that coverage was close or equal to 100% when using the augmented-coalescent likelihood for the true ω value of 0.1 and independent of $R_{0}$ or sample size (figure 2; supplementary material, figure S5). Coverage was lower when the true ω value was 0.9 (figure 2; supplementary material, figure S5). The augmented-coalescent likelihood was, in general, better when ω was small.

#### 3.1.2. Accuracy

For trees simulated with *Coalescent.jl* and *TiPS*, we observed that, in general, the estimates of α were very accurate showing lower values of relative error independent of sample size or true value of α. Note that when α was 2.94 or 9.65, the coalescent likelihood without augmentation produced very inaccurate results, showing relative error that was occasionally greater than 100-fold (figure 2; supplementary material, figure S5). For trees simulated with *diversitree*, accuracy was better for simulations in which the average $R_{0}$ was 1.22 and independent of sample size. However, accuracy was worse for estimating values of α=2.94 for lower $R_{0}$ and using the coalescent likelihood, and this was fixed by using the augmented-coalescent likelihood (figure 2; supplementary material, figure S5).

For trees simulated with *Coalescent.jl* and *TiPS*, we observed that when ω was set to the low value of 0.1, accuracy remained low and was unaffected by sample size. In addition, the use of the augmented-coalescent likelihood did not substantially improve the estimates. However, when ω was set to a high value of 0.9, the estimates remained highly accurate and unaffected by sample size (figure 2; supplementary material, figure S5). For trees simulated with *diversitree*, accuracy was better when the average $R_{0}$ was low. Furthermore, accuracy was slightly worse when ω was set to 0.1 (figure 2; supplementary material, figure S5).

Additional plots showing the estimated relative fitness parameters for individual replicates can be found in figures S6– S11 in the supplementary material. These figures span the range of tree simulators, numbers of tree tips and both likelihood methods.

### 3.2. Simulation and analysis of sequence alignments

**Figure 3.**
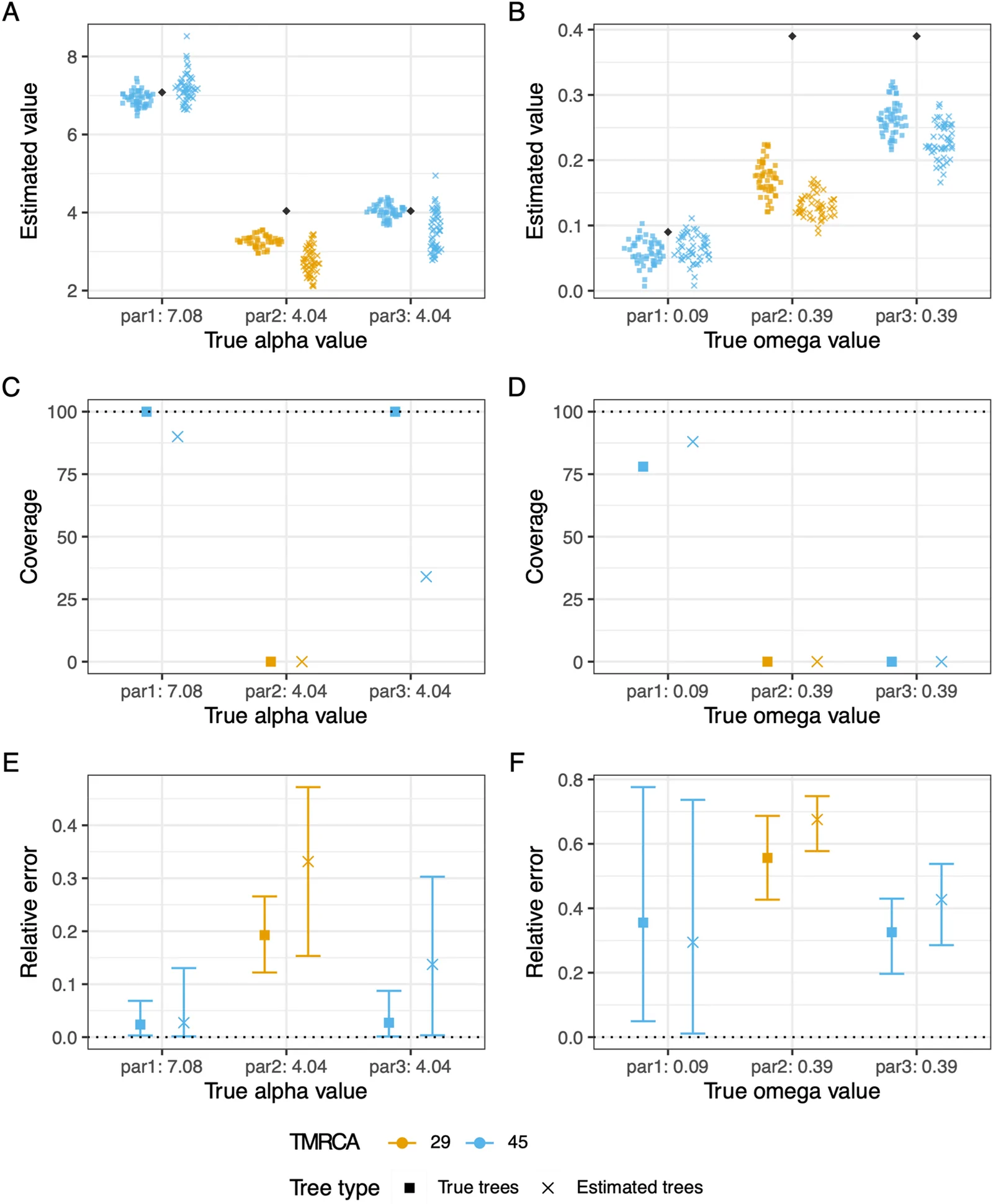
Performance in simulations with n=4056. Sina plots showing the true and estimated values for (A) α and (B) ω using the augmented-coalescent likelihood. Black diamond shapes in the plot are the true values. Coverage defined as the percentage of replicates that contained the true (C) α or (D) ω values within the 95% credible interval. Relative error for (E) α and (F) ω estimates. The relative error (y-axis) represents the computed median and the 0.025 and 0.975 quantiles for all replicates. All plots show the estimated values for the true *Coalescent.jl* trees and estimated time-scaled trees. The *par1*, *par2* and *par3* refers to the combination of parameter values 1, 2 and 3 (supplementary material, table S3). Sequence alignment length for *par1* was 2371 bp and for *par2* and *par3* was 105 bp. Fifty simulation replicates were generated for each combination of parameter values.

For analyses carried out with combination of parameters 1 (*par1*; supplementary material, table S3), we were able to estimate both α and ω using sequence alignments of 2371 bp. The estimated time-scaled trees produced results similar to those obtained with the true trees (figure 3). Furthermore, for *par1* (supplementary material, table S3), simulations were run for 45 years and the proportion of ancestral state reached 0.806, which was similar to the expected equilibrium value of 0.799 (supplementary material, table S3).

For analyses carried out with combinations of parameters 2 and 3 (*par2* and *par3*; supplementary material, table S3), we were unable to estimate α and ω using the true or estimated trees when the time to the most recent common ancestor (TMRCA) was 29 years. When the TMRCA was 45 years, we were able to estimate α using the true trees, but were unable to estimate ω with true or estimated trees (figure 3). However, when the simulations were run for long enough and the state proportions reached equilibrium, we could estimate both relative fitness α and ω (supplementary material, figure S4). Furthermore, for *par2* and *par3* (supplementary material, table S3), the proportion of ancestral state was 0.865 and 0.822, respectively, suggesting that it did not reach equilibrium (supplementary material, table S3). We were also unable to recover the true TMRCA and substitution rates with *treedater* using trees reconstructed with alignments of 105 bp (supplementary material, figure S3).

### 3.3. HIV-1 co-receptor tropism

The co-receptor tropism prediction of the subtype C PopART Phylogenetics sequences from Zambia revealed that 20.0% are capable of using the X4 co-receptor, associated with faster disease progression. This analysis showed that: (i) WebPSSM scores consistently split R5- and X4-capable viruses, (ii) there was a slight increase in the proportion of X4/R5X4 and decrease of R5 over the study period, (iii) the pre-treatment CD4 counts were similar for individuals infected by R5 and X4-capable viruses, and (iv) the proportion of X4/R5X4 tends to be higher for individuals in advanced stages of infection (low CD4) when compared with earlier stages (supplementary material, figure S13).

As expected given high within-host selective pressures for co-receptor switching, the fold-change in within-host replicative fitness (α) was much higher than one (6.87, 95% CI: 6.08–7.76) for the *env* analysis, while the fold-change in between-host replicative fitness (ω) was much lower than one (0.09, 95% CI: 0.04–0.18). These values are consistent with the hypothesis that the within-host relative fitness of variant (X4/R5X4) is substantially higher than the ancestral type (R5), while the between-host fitness of X4/R5X4 is much lower than R5. In other words, the magnitude of the transmission penalty of X4-capable viruses is approximately −90%.

These results were computed using the augmented-coalescent likelihood option, which demonstrated better performance with simulated data than the coalescent likelihood. Using the coalescent likelihood, we estimated notably different values for α (0.93, 95% CI: 0.67–1.28) and ω (0.38, 95% CI: 0.24–0.61). These are not as clearly consistent with the hypothesis but, given the performance of this likelihood option in our simulation studies with similar parameter values (figure 2; supplementary material, figure S12), we would not expect this option to produce reliable results.

## 4. Discussion

Pathogen fitness can be characterized at multiple levels corresponding to different life cycle stages. These include replication within target cells, immune evasion and the capacity to infect new hosts (transmissibility). At each stage, the selective pressures will be different, and they can conflict. For example, evolutionary changes to a pathogen that provide a fitness advantage within a host can potentially impair transmissibility between hosts. One way that transmissibility can be compromised is through increased pathogen virulence resulting from increased within-host fitness [1–4]. An example is HIV-1 transmission, which is governed by a transmission–virulence trade-off, where transmissibility is influenced by both replicative capacity and the duration of host survival [1–4]. Therefore, such multi-level selective pressures require multiple coefficients to describe fitness, but evolutionary analyses frequently collapse this complexity into a single measure [17,18].

We have developed a structured coalescent model which describes pathogen evolution of two co-circulating variants with multi-level (within- and between-host) selection. We further develop a ML method for inferring multi-level selection coefficients based on this model and a convenient software implementation. Our approach builds on previous work on related structured birth–death models (e.g. the BiSSE model) and adds new capabilities: our model improves on existing methods by accounting for nonlinear dynamics in effective population size, variable sampling through time, and provides a novel integration of a sampling likelihood with the genealogical coalescent likelihood. Our developments expand the applicability of this method, widely used in ecological studies, to epidemiological studies. In simulations, we find that the derived mutation–selection balance used in combination with the coalescent likelihood provides superior estimates of multi-level selection parameters. We further demonstrate that within-host selection coefficients can be estimated from population-based samples across a large range of values, from minor fitness costs to highly deleterious phenotypes. This provides an original avenue for estimating fitness cost of common deleterious variants from epidemiological data, which have not traditionally been used for this purpose. While our focus has been on evolution of infectious disease pathogens, the computational framework is quite general, and could also be applied to study macro-evolution.

In our simulations, we also demonstrated that when the genetic sequence alignment was sufficiently large and informative (similar to the empirical *env* gene), and the proportion of ancestral state and variant had reached equilibrium, we were able to estimate the relative fitness ω and α (figure 3). Our empirical analysis of the evolution of HIV co-receptor tropism supports the commonly hypothesized reduction in transmission associated with the X4-tropic virus and contra-indicates the ‘random transmission’ hypothesis [15,16] according to which both X4 and R5 tropic virus are transmitted equally in proportion to their viral titre. Our study appears to be unique in estimating a specific value for the fitness cost. This analysis, performed using data from a real and high HIV prevalence setting, confirmed that, when compared to R5, the within-host replicative fitness (α) of X4/R5X4 variants was higher (7-fold increase) and the between-host fitness (ω) was much lower (≈−90%). This estimate suggests that there are selective forces acting against X4 transmission and provides a magnitude for this transmission penalty [13,14]. Therefore, the lower frequency of X4 viruses in genital secretions is not the only factor acting against their transmission, although it is still very possible that X4/R5X4 variants will be transmitted if they dominate the donor's virus population, consistent with X4-tropic virus being transmitted at 9% of the rate of R5-tropic virus. The α estimates demonstrate pronounced within-host selection acting on X4/R5X4.

There are several limitations in our study. First, we used a single value for the infection generation time. However, it is possible that for the co-receptor analysis, infections with different co-receptors may lead to different rates of disease progression. Second, current state-of-the-art bioinformatic methods for the classification of co-receptor usage do not distinguish between the dual-receptor R5X4 and the single-receptor X4. Consequently, we were unable to test whether dual-receptor usage would provide any advantage in replicative fitness compared to X4 usage only.

Our model requires that the variants are in mutation–selection balance, and the simulation results reported in §3.2 show that performance in estimating parameters is negatively impacted when variants are not in equilibrium. A simple heuristic for determining whether the criteria for mutation–selection balance is met would be to plot the sampled variant proportions against time.

Our analysis of simulated sequence alignments of different lengths, also reported in §3.2, showed that it is possible to estimate the multi-level fitness parameters for sequence alignments of length similar to HIV-1 *env* gene (2371 bp) but not for those similar in size to the much smaller *V3 loop* (105 bp) region. Our expectation is that the information required to successfully estimate fitness parameters is not only a function of sequence length but also of mutation rate. While we have not sought to define the thresholds in these variables at which fitness parameter estimation becomes possible, further simulations emulating an empirical dataset of interest can be performed in order to check the applicability of our method.

In addition, our model assumes that within-host genetic diversity is small relative to between-host diversity. When this is not the case, it can cause divergence between the modelled and true transmission histories in three ways [45]: (i) internal nodes of the phylogenetic tree can predate transmission times [46], reducing the accuracy of transmission timings and increasing the possibility of incomplete transmission chains, (ii) when a donor transmits to more than one recipient, it is possible that the second recipient receives an older lineage [47], and (iii) more than one lineage can potentially be transmitted from donor to recipient, i.e. an imperfect ‘bottleneck’. With regard to HIV-1, the transmission bottleneck is narrow with typically a single lineage being transmitted [2,48]. However, these mitigating characteristics of HIV-1 may not be present for other pathogens and should be given further consideration when applied to other pathogens.

We have shown that our method is able to quantify the difference in fitness for two variants experiencing strong selective pressure. Our method is unlikely to be able to resolve fitness differences between many variants experiencing only weak selection. However, when strong selection exists, there is potential for this method to be expanded to incorporate more than two competing variants, such as adaptation driven by differing human leukocyte antigen (HLA) types in a host population. For example, the methods used by Palmer *et al.* [49] could be used to identify nucleotide sites that are linked to evolutionary pressure from HLA type and treatments that lead to drug resistance mutations, thereby allowing the definition of multiple phenotypes for analysis using the model presented here. While in its current form we expect there to be an issue with statistical identifiability as the number of parameters in the model is increased, this is an area for future development. Other developments may be possible related to the incorporation of more than one sequence per host, which if available could greatly enhance within-host selection estimates, as well as the incorporation of additional metadata (e.g. structural data or replicative capacity assays) when estimating selection coefficients.

## Supplementary Material



## Data Availability

The method used here is available as open source R package musseco at http://github.com/emvolz-phylodynamics/musseco and archived on Zenodo [43]. Scripts used to simulate and analyse data are available at http://github.com/thednainus/musseco_simulations and archived on Zenodo [50]. Scripts to analyse empirical data from HIV-1 are available at http://github.com/vinibfranc/BiSSECo_coreceptor_PANGEA and archived on Zenodo [51]. Access to the PANGEA PopART HIV-1 sequence dataset is through application. Details are available at http://www.pangea-hiv.org/join-us. Supplementary material is available online [52].

## Appendix A. PANGEA consortium members

Christophe Fraser${}^{1}$, Kate Grabowski${}^{2}$, Sikhulile Moyo${}^{3}$, Deenan Pillay${}^{4}$, Andrew Rambaut${}^{5}$, Oliver Ratmann${}^{6}$, Deogratius Ssemwanga${}^{7}$, Lucie Abeler-Dörner${}^{1}$, Helen Ayles${}^{8}$, David Bonsall${}^{1}$, Rory Bowden${}^{1}$, Max Essex${}^{9}$, Sarah Fidler${}^{10}$, Tanya Golubchik${}^{1}$, Ravindra Gupta${}^{11}$, Richard Hayes${}^{12}$, Joshua Herbeck${}^{13}$, Joseph Kagaayi${}^{14}$, Pontiano Kaleebu${}^{7}$, Jairam Lingappa${}^{13}$, Vladimir Novitsky${}^{15}$, Thomas Quinn${}^{2}$, Janet Seeley${}^{7,16}$, Frank Tanser${}^{17}$, Maria Wawer${}^{2}$, Myron Cohen${}^{18}$, Vincent Calvez${}^{19}$, Thumbi Ndung'u${}^{17}$, Tulio D'Oliveira${}^{20}$, Ann Dennis${}^{18}$, Dan Frampton${}^{4}$, Anne Hoppe${}^{4}$, Paul Kellam${}^{11}$, Cissy Kityo${}^{21}$, Andrew Leigh-Brown${}^{5}$, Nick Paton${}^{22}$.

${}^{1}$ University of Oxford, Oxford, UK, ${}^{2}$Johns Hopkins University, Baltimore, USA, ${}^{3}$Botswana Harvard AIDS Institute Partnership, Botswana, ${}^{4}$University College London, London, UK, ${}^{5}$University of Edinburgh, Edinburgh, UK, ${}^{6}$Imperial College London, London, UK, ${}^{7}$Medical Research Council/Uganda Virus Research Institute, Entebbe, Uganda, ${}^{8}$PopART/Zambart, Lusaka, Zambia, ${}^{9}$Harvard Botswana, Gaborone, Botswana, ${}^{10}$PopART/Imperial College London, UK, ${}^{11}$University of Cambridge, Cambridge, UK, ${}^{12}$PopART/London School of Hygiene and Tropical Medicine, London, UK, ${}^{13}$University of Washington, Seattle, USA, ${}^{14}$Rakai Health Sciences Program, Kalisizo, Uganda, ${}^{15}$Harvard University, Boston, USA ${}^{16}$London School of Hygiene and Tropical Medicine, London, UK, ${}^{17}$Africa Health Research Institute, South Africa, ${}^{18}$University of North Carolina, USA, ${}^{19}$Institut Pasteur, Paris, France, ${}^{20}$University of KwaZulu-Natal, South Africa, ${}^{21}$EARNEST/The Joint Clinical Research Centre, Kampala, Uganda, ${}^{22}$EARNEST/University of Singapore, Singapore.
